# Bibliometric Analysis of Literature Published on Antibacterial Dental Adhesive from 1996–2020

**DOI:** 10.3390/polym12122848

**Published:** 2020-11-29

**Authors:** Abdul Samad Khan, Shafiq Ur Rehman, Yara Khalid AlMaimouni, Shakil Ahmad, Maria Khan, Murtaza Ashiq

**Affiliations:** 1 Department of Restorative Dental Sciences, College of Dentistry, Imam Abdulrahman Bin Faisal University, Dammam 31441, Saudi Arabia; ykalmaimouni@iau.edu.sa; 2 Deanship of Library Affairs, Imam Abdulrahman Bin Faisal University, Dammam 31441, Saudi Arabia; suRehman@iau.edu.sa; 3 Central Library, Prince Sultan University, Riyadh 11586, Saudi Arabia; shakil@psu.edu.sa; 4 Department of Oral Biology, University of Health Sciences, Lahore 54000, Pakistan; mariakhan1685@gmail.com; 5 Islamabad Model College for Boys, H-9, Islamabad 44000, Pakistan; gmurtazaashiq00@gmail.com

**Keywords:** dental adhesives, antibacterial adhesives, bonding agents, bibliometric analysis, mapping review, Web of Science, healthcare

## Abstract

This study aimed to investigate the current state of research on antibacterial dental adhesives. The interest in this field can be drawn from an increasing number of scholarly works in this area. However, there is still a lack of quantitative measurement of this topic. The main aim of this study was to consolidate the research published on the antibacterial adhesive from 1996 to 2020 in Web of Science indexed journals. The bibliometric method, a quantitative study of investigating publishing trends and patterns, was used for this study. The result has shown that a gradual increase in research was found, whereby a substantial increase was observed from 2013. A total of 248 documents were published in 84 journals with total citations of 5107. The highly cited articles were published mainly in Q1 category journals. Most of the published articles were from the USA, China, and other developed countries; however, some developing countries contributed as well. The authorship pattern showed an interdisciplinary and collaborative approach among researchers. The thematic evaluation of keywords along with a three-factor analysis showed that ‘antibacterial adhesives’ and ‘quaternary ammonium’ have been used commonly. This bibliometric analysis can provide direction not only to researchers but also to funding organizations and policymakers.

## 1. Introduction

Adhesive dentistry is an integral part of the contemporary aesthetic restoration, and it is constantly evolving to promote conservative dentistry [1]. The polymer-based adhesive restorations have been extensively used along with five hundred million composite restorations per year [2]. The history of dental adhesives started in 1949 with the concept of adhesion to dentin through physical and chemical bonds. In 1954, the first experiment on adhesion to enamel was successfully done with acid etching [3,4]. With advancing technologies, new generations were introduced from non-etch to total-etch (fourth and fifth generation) to self-etch universal adhesives (eighth generation) systems. One promising advancement includes the addition of novel biomaterials that can effectively regulate mineral deposition in dentin and promote remineralization [5]. Modifying adhesives to improve the chemical stability of resin-based restoration have shown resistance against esterase and water degradation. For instance, formulating a bioadhesive modified with catechol groups such as synthetic mussel biomimetic polymers can significantly improve shear bond strength and water-resistance properties [6]. Moreover, adding antimicrobials and enzymatic inhibitors can reduce biofilm formation and penetration within adhesive interfaces [7]. 

The adhesive interface should remain stable over time to provide clinical durability for dental restorations with proper marginal seal. However, all adhesive systems are susceptible to degradation [8]. Interaction with active bacterial enzymes, dental biofilm, and various enzymes can lead to chemical breakdown [1]. Subsequently, degradation of interfaces may lead to adhesive failure causing microleakage, separation, and micro-movement between materials [9]. Microleakage at the dentin-adhesive interface can allow the growth and accumulation of bacterial biofilm, which causes dental caries [10,11]. The addition of antibacterial agents in dental restoration systems can interfere with the growth and reproduction of residual bacteria and prevent the invasion of new bacteria [12]. Thus, reducing secondary caries occurrence and improving the longevity of dental restorations [13]. It is estimated that a total of $298 billion is spent globally every year for the replacement of failed restorations; this represents an average of 4.6% of the total global healthcare related expenditures [14].

Antibacterial agents can be leachable compounds, polymerizable monomers, and filler particles [15]. Several studies have been conducted to evaluate the effect of adding nanoparticles such as silver, calcium phosphate, zinc oxide, and titanium dioxide in various restorative materials, including glass ionomer cements, resin-based materials, and dental adhesives [16,17,18]. The quaternary ammonium compounds (QAC) have been used in composite restorations and impact the polymeric structure, degree of conversion, solvent sorption, shrinkage, and alter the biocompatibility behavior [19]. It is reported that the antibacterial activity of QAC containing restorative materials, were adversely altered due to electrostatic interaction between saliva and QAC [20]. The addition of antibacterial agents in adhesive systems helps to improve the stability of dentin–resin interface that is essential for the durability of restorations [21,22,23]. Antibacterial components’ addition can positively affect interfacial bonding [24], decrease bacterial viability [25], inhibit matrix metalloproteinase, and most importantly, it reduces the effect of causative factors initiating chemical degradation of dental adhesives [22,26]. The antibacterial dental adhesive is the primary restorative material with the ability to restrict or inhibit secondary caries. In the last few years, significant interest related to antibacterial dental adhesives has been noticed, and few literature review articles have been published on the use of antibacterial monomers and nanoparticles in dental adhesives [19,27,28]. However, it is essential that the studies related to this topic should be analyzed so that their impact on applied research in the field of adhesive dentistry can be evaluated. One of the methods to evaluate the impact in applied science can be through bibliometric analysis. Bibliometric analysis has been performed in various sub-specialties of dentistry including periodontology [29], oral and maxillofacial surgery [30], implantology [31], orthodontics [32], prosthodontics [33], pediatric dentistry [34], and endodontics [35]. However, the authors could not find any bibliographic study where studies on antibacterial dental adhesive were assessed quantitatively. Therefore, in this study, various aspects were considered to assess the publications and citation trends in antibacterial dental adhesive from 1996–2020. The other research questions of this study were:

1. Which are the leading countries and institutions in antibacterial dental adhesive literature?
2. Which are the most influential journals in antibacterial dental adhesive literature?
3. Which are the most trending and cited publications of antibacterial dental adhesive literature?
4. What are the authorship and collaboration research patterns of antibacterial dental adhesive researchers?
5. What are emerging research themes/keywords in antibacterial dental adhesive literature?

## 2. Methodology

**Figure 1:**
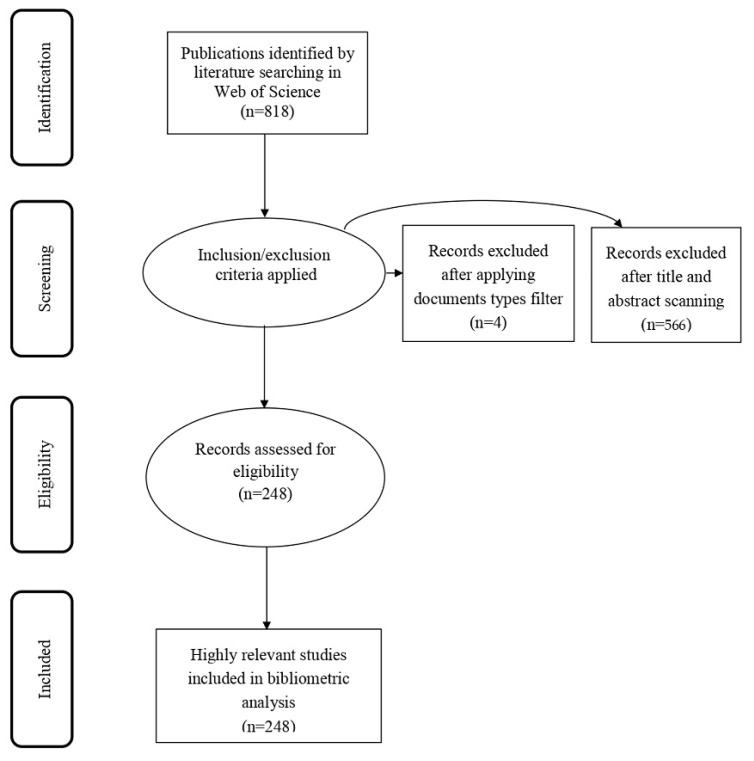
Four-phase flow diagram of data extraction and filtration process of dental adhesive publications.

The bibliometric study investigates the publishing trends of antibacterial dental adhesive literature indexed in Web of Science (WOS) core collection database. It is recognized that WOS is the most authentic and reliable indexing and abstracting database globally, whereas bibliometric analysis is a statistical method to analyze the publishing trends, patterns, and scope of the published scholarly work. A comprehensive four-phased search and selection strategy was framed, as shown in Figure 1, to include publications to conduct bibliometric analysis. The following query was designed, adding all the relevant keywords, to run into Web of Science core collection to retrieve the results. Boolean operators were used to combine the keywords to retrieve the maximum and relevant results. (TS = (dental adhesive AND antibacterial OR dental bonding agent AND antibacterial OR dental adhesive AND antibacterial activity OR dental adhesive AND biofilm OR antibacterial dental adhesives OR dental adhesive AND bacteria OR dental adhesive AND quaternary ammonium derivative OR dental adhesive AND antibacterial monomers OR dental adhesive AND anti-bacterial activity OR dental adhesive AND antibacterial effect OR dentin bonding AND antibacterial effect OR dental adhesive AND silver OR dental adhesives AND antibacterial agent OR dental adhesive AND antibacterial particles)).

### 2.1. Search Strategy

For inclusion and recall of maximum relevant records, a WOS core collection field (it has over 21,000 peer-reviewed journals published worldwide) has been selected, and further, the “Advanced Search” facility of WOS core collection database was chosen. There was no filter applied (language and timespan) while retrieving the results/records. A total of 818 records appeared, which were downloaded on 7 July 2020. 

### 2.2. Inclusion and Exclusion Criteria

For the precision purpose, inclusion and exclusion criteria were applied to refine the results further. 

Inclusion Criteria: The authors did not find any irrelevant records to exclude at the eligibility stage. Two hundred and forty-eight (248) records were assessed to process at the eligibility stage, and these records obtained a total of 5107 citations. The included document types were articles (234 articles obtained 5033 citations), review articles (7 articles obtained 46 citations), proceeding papers (6 documents obtained 28 citations), and early access (one document with no citation). 

Exclusion Criteria: For this purpose, the filter of “document types” was applied and only “articles, review articles, proceeding papers, and early access” were retrieved. This purification process helps to remove four documents. To ensure the accuracy and relevancy of the data, each record was thoroughly read (title and abstract), and 570 irrelevant and duplicate records were excluded from the data. 

### 2.3. Data Analysis

Finally, 248 highly relevant records [10,24,25,36,37,38,39,40,41,42,43,44,45,46,47,48,49,50,51,52,53,54,55,56,57,58,59,60,61,62,63,64,65,66,67,68,69,70,71,72,73,74,75,76,77,78,79,80,81,82,83,84,85,86,87,88,89,90,91,92,93,94,95,96,97,98,99,100,101,102,103,104,105,106,107,108,109,110,111,112,113,114,115,116,117,118,119,120,121,122,123,124,125,126,127,128,129,130,131,132,133,134,135,136,137,138,139,140,141,142,143,144,145,146,147,148,149,150,151,152,153,154,155,156,157,158,159,160,161,162,163,164,165,166,167,168,169,170,171,172,173,174,175,176,177,178,179,180,181,182,183,184,185,186,187,188,189,190,191,192,193,194,195,196,197,198,199,200,201,202,203,204,205,206,207,208,209,210,211,212,213,214,215,216,217,218,219,220,221,222,223,224,225,226,227,228,229,230,231,232,233,234,235,236,237,238,239,240,241,242,243,244,245,246,247,248,249,250,251,252,253,254,255,256,257,258,259,260,261,262,263,264,265,266,267,268,269,270,271,272,273,274,275,276,277,278] on antibacterial dental adhesive were selected for data analysis and visualization using various bibliometric applications and software, including MS Excel, VOSViewer, Biblioshiny (RStudio), CiteSpace, and BibExcel.

### 2.4. Terms Used in Data Analysis

Some terms/abbreviations are used for data analysis in various columns of tables as TP stands for total publications, TC for total citations, IF for impact factor, Q for journal quartile, and FY for the first year of publication on this specific topic/area. The citation impact describes the average citation received by a specific publication. The citation impact (CI) in this study was calculated by dividing the total number of citations by the total number of publications. This illustrates the average number of citations that a specific publication has received [279]. In addition, there are some terminologies defined by Web of Science database that were used as U1, U2, and Z9. U1 refers to the usage count of the last 180 days. Usage count is briefed by Web of Science as “the count of the number of times the full text of a record has been accessed, or a record has been saved in the last 180 days. This count can move up or down as the end date of the fixed period advances” [280]. Since the data was retrieved on 19 July 2020, so the last 180 days begin from this date. Similarly, U2 is the usage count since 2013, and Z9 is the total times cited count from (Web of Science Core Collection, Arabic Citation Index, BIOSIS Citation Index, Chinese Science Citation Database, Data Citation Index, Russian Science Citation Index, SciELO Citation Index). Moreover, the usage count is updated on a daily basis [280]. 

## 3. Results

### 3.1. Analysis of the Overall Growth Trend

**Figure 2:**
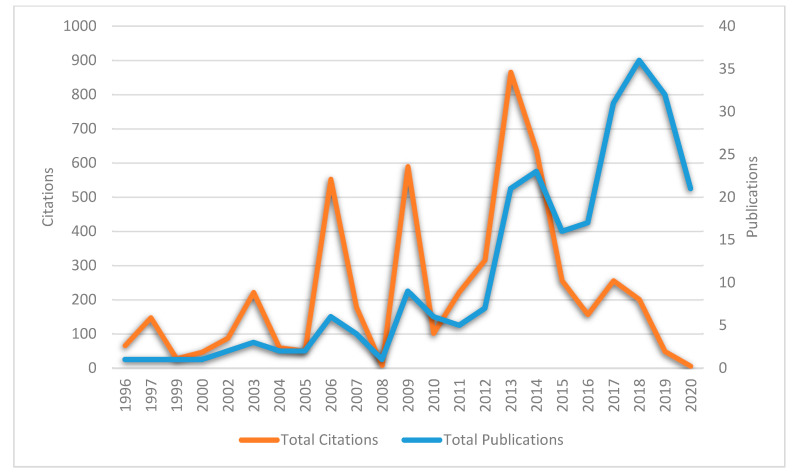
Publishing and citation trends in antibacterial dental adhesive research.

Figure 2 presents the total number of publications and citations on dental adhesive literature. It shows that this area has grown tremendously in the last couple of years of the 20th century. The first few years have observed little progress as the first publication appeared in 1996. Four publications in the first five years (1996–2000) have obtained 289 citations; however, the growth has been increasing each year gradually. The notable publications have been indexed from 2013 onwards, whereas the citations have been gradually increasing annually. The boom of the publications and citations was 2018 (TP = 36) and 2013 (TC = 866), respectively. 

### 3.2. Most Productive Countries and Organization on Antibacterial Dental Adhesive Research

Table 1 presents the top 10 most productive countries on dental antibacterial adhesive literature globally. There are five countries having publications in double-digits, and three countries produced over 50 publications individually. The United States of America emerged as a top country with the highest publications (99), citations (2480), and total link strength score (1202), followed by China with 91 publications and 2473 citations and Brazil with 59 publications and 861 citations. It is interesting to highlight that Spain is at the bottom of the table with only six publications, and Japan is ranked fourth; however, their publications gain the highest citation impact, 46.83 and 41.68, respectively.

Among the top 10 organizations, the University of Maryland produced 53 publications and 1713 citations, followed by Sichuan University with 32 publications and 1128 citations. It is worth mentioning that Osaka University is at the sixth rank (16 publications, 810 citations) but has the highest citation impact (CI = 50.63).

### 3.3. Highly Influential Journals

Table 2 records the highly productive top ten journals on dental antibacterial adhesive literature. Most of the sources have produced 10 and fewer than 10 publications. The source *Dental Materials* is on the top of the list with a remarkable number of publications (40), citations (1468), citation impact (36), fall in Quartile 1 and has impact factor 4.5 followed by *Journal of Dentistry* at 2nd rank with 29 publications, 747 citations, 25.76 citation impact, Quartile 1 and with impact factor 3.24. The source with the highest impact factor *Acta Biomaterialia* (7.24) has produced 10 publications and obtained 312 citations. All included sources are impact factor from Quartile 1–3 and half of them belong to The Netherlands, four from the United States of America, and one from Japan. 

### 3.4. Authorship Pattern

**Figure 3:**
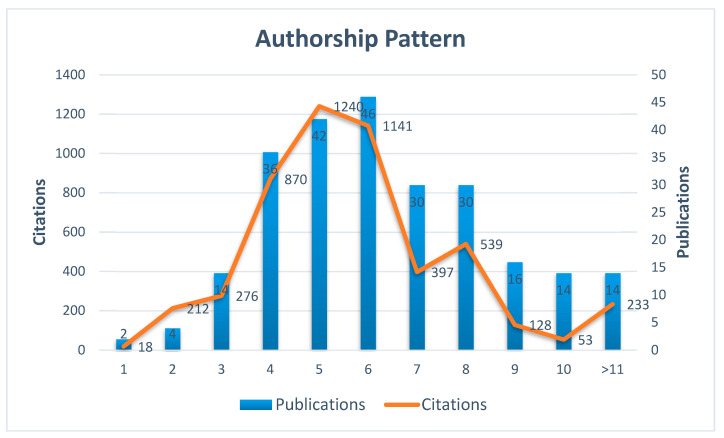
Authorship pattern of antibacterial dental adhesive literature.

Figure 3 describes the authorship pattern on dental antibacterial adhesive literature. The authorship pattern ranges from a minimum of one author to a maximum of 15 authors. There are only two publications as single-author, four publications as two-authors, and 14 publications as three-authors. It shows the trend of collaborative research pattern in this area has the highest numbers of publications (46) that were produced by six-authors, 42 produced by five-authors, 36 produced by four-authors, and 30 publications by seven and more-authors. There are 14 publications with more than 10 authors. Overall, the authorship patterns inform that collaborative studies (more than three-authors) are being implied in dental antibacterial adhesive literature worldwide.

### 3.5. Authors’ Keyword Analyses on Antibacterial Dental Adhesive 

**Figure 4:**
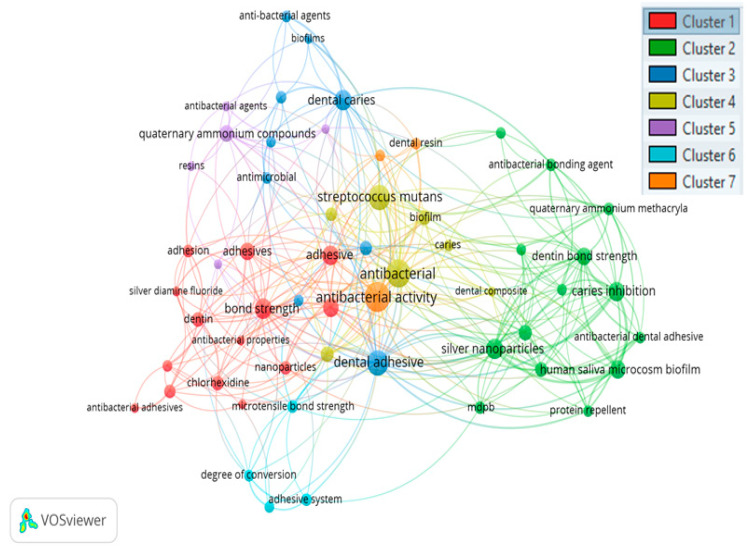
Co-occurrence network of author keywords (minimum number of occurrences: three). A co-occurrence network is a relationship of two or more keywords occurring together. In this figure, there are seven clusters (indicating various colors) having relationship with each other. Cluster one is the strongest network relationship, followed by 2–7 clusters, respectively. The top keyword ‘antibacterial’ having the strongest relationship and occurred maximum in antibacterial dental adhesive literature.

Figure 4 presents the authors’ keyword analysis on dental antibacterial adhesive literature. Three minimum number of occurrences of a keyword were selected; hence, out of total 587 authors’ keywords, 83 meet this criterion consisting of seven clusters. Each color represents a separate cluster and clusters are arranged on the basis of link strength and occurrence. Hence, the size of the bubble indicates the nature of the relationship with link strength and occurrence. The five keywords with the highest total link strength are antibacterial (link strength: 59), dental adhesive (55), antibacterial activity (52), caries inhibition (50), and streptococcus *mutans* (50).

### 3.6. Thematic Evolution Map of Author Keywords 

**Figure 5:**
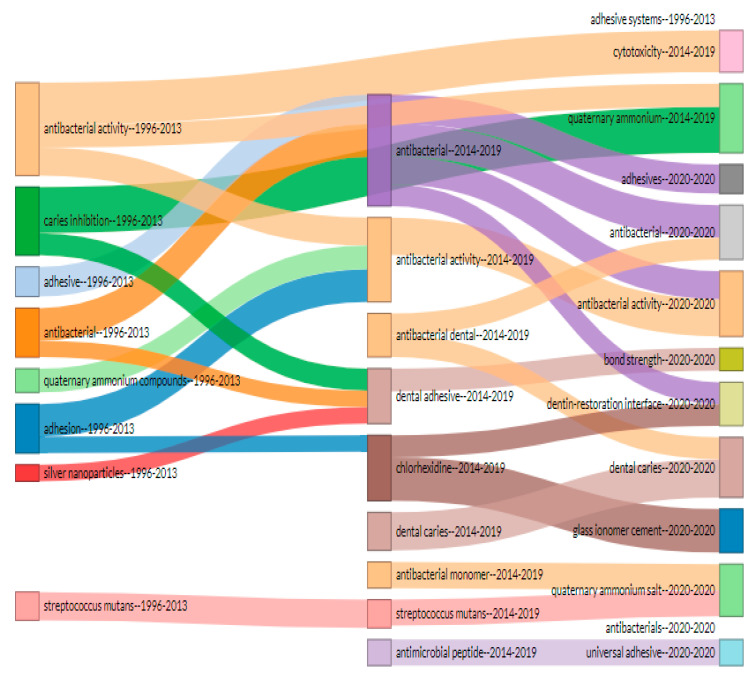
Thematic evolution map of keywords from 1996 to 2020 with respect to antibacterial dental adhesives. This map highlights the usage and emergence of various keywords related to antibacterial dental adhesive literature. It is evident that antibacterial activities, adhesive, antibacterial, quaternary ammonium were important keywords throughout the 25 years (1996–2020). Few keywords—such as chlorhexidine, antimicrobial peptides, antimicrobial monomers, etc.—emerged from 2014.

The thematic evolution of keywords during the last 25 years shows a clear shift in antibacterial dental adhesive research streams. Caries inhibition, adhesion, silver nanoparticles, related keywords disappeared after 2013 (Figure 5). The results show that antibacterial dentistry, chlorhexidine, dental caries, antibacterial monomers, and antimicrobial peptides were hot topics from 2014 to 2020. In addition, antibacterial activities, adhesive, antibacterial, quaternary ammonium were important keywords throughout the 25 years (1996–2020).

### 3.7. Highly Cited Articles on Antibacterial Dental Adhesive

Table 3 presents the bibliographic information of the top ten highly cited articles on dental antibacterial adhesive. The top 10 highly cited articles’ citations and years ranged a maximum of 300 to 95 citations and 1997 to 2013, respectively. There are two articles that obtained over 150 citations. The article titled “Antibacterial Activity of Dental Composites Containing Quaternary Ammonium Polyethyleneimine Nanoparticles Against *Streptococcus* *mutans*” by Beyth N, published in *Biomaterials* in the year 2006, is on the top of the list with the highest citations (300), average citation (21.43), followed by an article written by Imazato S. that obtained 181 citations. Interestingly, more than half of the highly cited articles were published in *Dental Material Journal,* and this journal is also on the top of the list of most influential sources (Table 2). 

### 3.8. Three Factor (Sources, Countries, and Keywords) Relationships 

**Figure 6:**
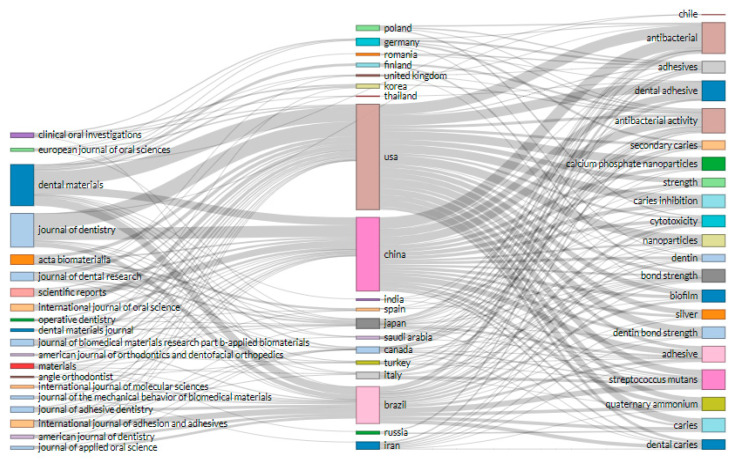
Three-factor plots of the relationship among keywords (left), countries (middle), and sources (right) on antibacterial dental adhesive literature. This figure shows that the researchers from top contributing countries (USA, China, and Brazil) prefer to publish their work in ‘dental material’ and ‘journal of dentistry’ with keywords like ‘antibacterial’, ‘antibacterial activity’, ‘dental adhesive’. Most probably, in bibliometric studies, the top indicators include influential countries, sources, keywords, and authors. This figure shows that researchers from various countries prefer to publish their work in specific sources and with preferable keywords. The largest block shows a greater connection and relationship.

Figure 6 presents the three-factor analysis on the relationship among sources (left), countries (middle), and keywords (right) to better understand which countries prefer to publish with which keywords and in what sources. The relationship indicates that three top countries (USA, China, Brazil) have a strong relationship with six sources (*Dental Material*, *Journal of Dentistry*, *Acta Biomaterialia*, *Journal of Dental Research*, *Journal of Biomedical Material Research*, *International Journal of Adhesion and Adhesive*) and prefer to publish with eight keywords (antibacterial, antibacterial activity, dental adhesive, streptococcus *mutans*, adhesive, quaternary ammonium) respectively. 

### 3.9. Collaboration World Map of Dental Antibacterial Adhesive Literature

**Figure 7:**
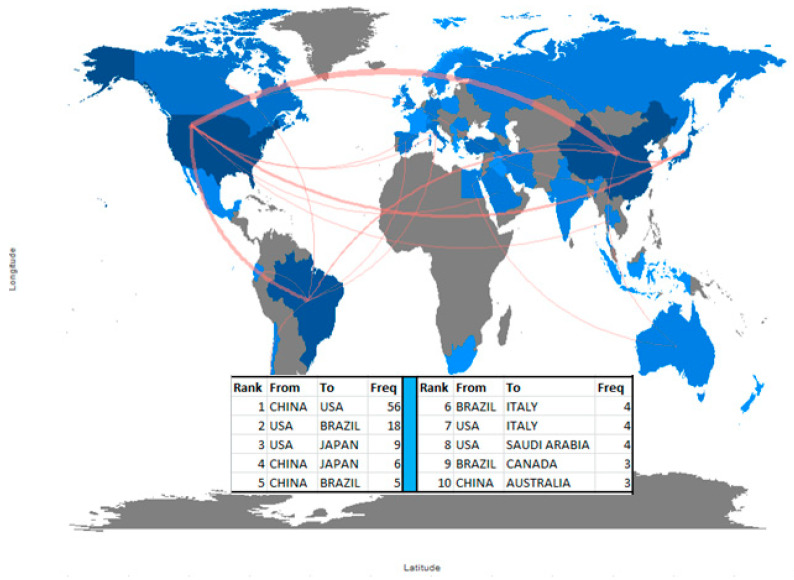
Country collaboration map on dental adhesive literature.

Figure 7 describes the country collaboration map of dental antibacterial adhesive literature. There are a total of 56 collaboration entries recorded worldwide. The United States of America, China, and Brazil were the major collaborators with other countries worldwide. The Table in Figure 6 indicated the top ten collaborations show that major two collaborations have been between China and the USA (56) and the USA and Brazil (18). The rest of the 54 collaboration entries are in a single digit. Most of the collaborations’ occurrence is one and two collaborations between various countries. There are 35 single collaborations (one collaboration) and 11 bi-collaborations (two collaborations). The remaining top 10 collaborations are highlighted in Figure 7.

### 3.10. Citation Bursts of Dental Adhesive Literature 

**Figure 8:**
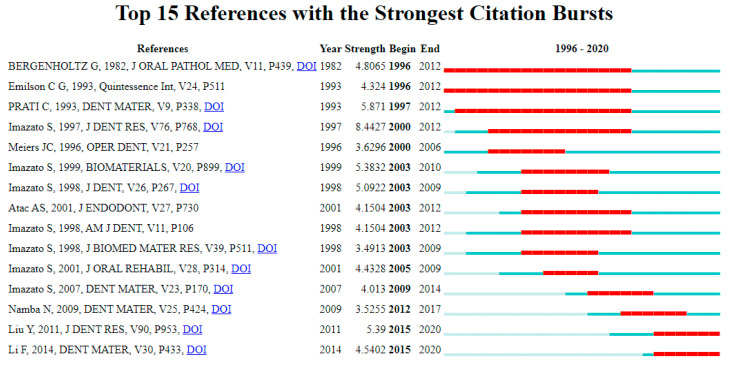
Citation bursts of dental adhesive articles (1996–2020).

Figure 8 describes the top 15 references of dental adhesive literature with the strongest citation bursts. These citation bursts expanded between the years 1996–2020. Most of the references ranged between the digits 3.00 to 5.00 as the strongest citation bursts. Albeit, the two citations on the top have the most expanded duration (1996–2012); however, the strength of their citation bursts is low (4.80 and 4.32), respectively. The reference of work written by Imazato S. in 1997 had the highest citation burst (8.44) and expanded between the years 2000–2012, whereas the reference with the least strength of citation bursts (3.49) was also written by Imazato S. in 1998. 

## 4. Discussion

The bibliometric analyses have been widely used methods for studying the structures and mechanisms of science. In this study, the qualitative evaluation of scientific publications as the direct output of research was performed using various variables (research questions), which are discussed below. 

### 4.1. Yearly Trend of Publications and Citations

The bibliographic search in this study showed that the first paper related to this topic, i.e., the antibacterial dental adhesive, was published in 1996; however, no specific antibacterial agent was incorporated in the dental adhesive. Therein, the authors claimed that antibacterial activity was due to the presence of glutaraldehyde in the formulation. In the first four years, only one paper was published each year on this topic, whereby MDPB as antibacterial monomer was introduced in the dental adhesive in 1997 by Imazato and group. This paper was published in Journal of Dental Research (Q1) and got 258 citations, and was rated as the fourth most-cited article. The authors claimed that the knowledge of the effect of incorporating different concentrations of MDPB in dentin primer would help the clinicians and scientists to improve the performance of dental adhesives. 

### 4.2. Highly Productive and Cited Countries and Organizations

The United States has the largest number of publications and citations. The scientific contribution of the USA to dental research and development occurred due to many factors; these include the establishment of the National Institute of Dental Research in 1948, funding opportunities from both government and private sectors, and the formation of scientific journals [281]. China was found to have the second-largest number of publications. This rise in publications from both countries can be attributed to the high frequency of collaboration between academic institutions in China and the USA. In addition, the top five influential organizations were from the USA and China, where the University of Maryland, USA, has the largest number of publications followed by three Chinese institutions. 

### 4.3. Highly Cited Papers

Later, in the early 2000s (2002, 2003, and 2006), papers based on MDPB antibacterial adhesives were published by the same group and received citations. The paper published in *Dental Materials* (Q1) in 2006 by this group got fourth-highest citations (257). In 2006, Beyth et al. [55] utilized quaternary ammonium polyethyleneimine nanoparticles (1 wt %) as an antibacterial agent in adhesives. The work was published in Biomaterials (Q1) and is the most-cited article (300 citations). The material was synthesized by cross linking PEI with dibromopentane monomer. The in vitro antibacterial testing showed no inhibition zone in the agar test, however, in direct contact test and eluted components the antibacterial ability of experimental adhesive was strongly evident even after 1 month of aging. 

The second most-cited article by Imazato et al. [137] was published in the year 2003 (181 citations). This article was published in *Dental Materials* (Q1) and examined the antibacterial efficiency of 2.5% 12-methacryloyloxydodecylpyridinium bromide (MDPB) monomer in resin-based adhesive. The results showed that the inhibition zone was significantly higher in the experimental adhesive by a mean level of 97%. The study confirmed the antibacterial ability of the modified resin adhesive without adversely affecting the bond strength or degree of conversion. 

The third most-cited article is by Imazato et al. [138] published in *Journal of Dental Research* (Q1) in the year 1997 (148 citations), which assessed the antibacterial efficacy of incorporating 1, 2, and 5% of MDPB into dentin primer against S. *mutans*, *Actinomyces viscosus*, and Lactobacillus bacteria. The results of 5% MDPB incorporation showed complete bactericidal effects against S. *mutans* in 30 s with a similar effect on Actinomyces *viscosus*, and higher effect on Lactobacillus. The article also found no adverse effect on tensile bond strength and degree of conversion. The knowledge of the effect of incorporating different concentrations of MDPB in dentin primer helps clinicians and scientists to improve the performance of dental adhesives.

The fourth most-cited article was published in 2009 (*Dental Materials*: Q1) by Ahn et al. [36] (146 citations), which assessed the cariogenic streptococci adhesion, growth, and diffusion after adding specific wt. % of silica nanofillers and various ppm concentrations of silver nanoparticles into composite adhesive. The adhesion and growth of S. *mutans* were lower in experimental groups compared with conventional adhesives. At the same time, no inhibition zones were observed among the groups after 48 h. 

The fifth most-cited article was again published in *Dental Materials* (Q1) by Imazato et al. in 2006 (138 citations). The authors have compared the Clearfil Protect Bond primer (PB) with three different modified materials (PB without MDPB, PB without MDPB, and 10-methacryloyloxydecyldihydrogen phosphate (MDP), and PB without MDPB/MDP and with 1% Cetylpyridinium chloride). Antibacterial efficacy was determined against S. *mutans*, Lactobacillus *casei*, and Actinomyces *naeslundii* through agar disc-diffusion test, determination of minimum inhibitory/bactericidal concentrations, and assessment on bacterial-impregnated demineralized dentin blocks. The PB primer was the most effective against bacteria compared with modified materials. The knowledge of the acidity effect on antibacterial efficacy when using MDPB-containing materials can help clinicians and scientists to develop a protocol of using the material in multiple applications.

Since 2013, the trend has been shifted to publish more papers about antibacterial adhesives. This study identified the main geographic areas in the world related to publications based on antibacterial adhesive studies production. It was found that the maximum number of publications came from the USA (39.4%), China (36%), Brazil (23.5%), and Japan (8.7%). The publications on antibacterial adhesives were also observed in emerging countries, such as Turkey (5%), Italy (3%), South Korea (2.3%), and Spain (2.3%). It is anticipated that the high GDP rate and expenditures on R&D might be a reason for more publications from the USA and China. The distribution of published articles shows the clear influence of economic and development levels. However, these reasons were not fully investigated in this study and beyond the scope. The growth in the number of publications in other countries such as Turkey and South Korea reflects the efforts being undertaken and their increasing presence in the international setting. The most prolific author in this area is Imazato, S. (Japan), and their four research papers are in the top 10 most cited papers, and the cumulative citation of these papers is 573. His seven papers also appeared in the top 15 strongest citation bursts that shows the interest of researchers in his area of research. 

### 4.4. Preferred Journals

The 4 out of 10 most preferred publication outlets for antibacterial adhesive publications are listed in the Q1 ranking of Web of Science; 3 of these are from the (Elsevier Sci. Ltd., Amsterdam, The Netherlands), and 1 is from the USA (Sage Publishing Inc, Thousand Oaks, CA, USA). *Dental Materials* is ranked first with 40 articles (15.9%). Similarly, the *Journal of Dentistry* and *Journal of Dental Research* have published 29 (11.5%) and 10 (3.98%) articles, respectively. *Dental Materials Journal* is last in the list of highly influential journals, and 5 (1.99%) articles were published in this journal. The trend is more in high impact factor journals, and most influential journals are in Q1 (4), Q2 (4), and Q3 (2) category. Out of these, an article by Imazato et al. (2003) is ranked second in the highly cited top 10 articles on antibacterial adhesives.

Similarly, six most cited articles were published in *Dental Materials* (Elsevier Sci. Ltd.), two in *Journal of Dental Research* (Sage Publishing Inc.), and one each in *Journal of Dentistry* (Elsevier Sci. Ltd.) and *Biomaterials* (Elsevier Sci. Ltd.). All these journals are from developed countries, follow rigorous peer-reviewing, and publish articles only in the English language. Secondly, the *Dental Materials*, *Journal of Dental Research*, and *Journal of Dentistry* are specialized journals from the Dentistry category. However, biomaterials is an interdisciplinary journal and thus publishes research from different disciplines, including biomedical materials and dentistry. 

### 4.5. Frequently Used Keywords and Emerging Keywords (1996–2013, 2014–2016)

The findings in this study showed that the keywords determined the trend of publications in this area. The most frequently used keywords would help researchers look for articles relevant to antibacterial adhesives. The three-factor relationship showed that the “antibacterial” keyword was mainly used by authors from the USA and China. This particular keyword seems to be generic; however, it was used repeatedly. Less attention has been given to keywords associated with inorganic fillers with antibacterial properties such as silver, silver diamine fluoride. Furthermore, no attention has been given to those inorganic components such as zinc, magnesium, and polymeric contents having antibacterial properties. However, highly cited papers are based on MDP and MDPB based adhesives. 

In thematic evaluation, it was found that keywords such as ‘antibacterial activities’, ‘antibacterial’, and ‘quaternary ammonium’ have been used throughout the 25 years. Similarly, ‘antibacterial monomer’ has been used frequently during 2014–2020. The use of these keywords might be due to the introduction of the concept of ‘immobilized bactericide’ in dentistry by Imazato et al. [282]. Therefore, the use of antibacterial compounds has gained interest to be used in dental adhesives. This innovative idea of developing antibacterial monomers and their use in composite resin was first given in 1993 [283]. Later, it was introduced in dental adhesives and bonding agents, and the material was commercialized (Clearfil Protect Bond, Kuraray Medical Inc, Okayama, Japan). Another keyword, which has been used frequently is ‘chlorhexidine’. Many studies have shown promising results using this material and considered chlorhexidine as the most effective antibacterial material. In contrast to this, surprisingly, ‘silver nanoparticle’ disappeared after 2013, which could be due to discoloration of tooth structure caused by silver particles [284].

### 4.6. International Collaboration and Authorship Patterns 

Various national and international programs have been launched in many countries to focus on promoting interdisciplinary research through research funding and grant [285]. It has become increasingly important that oral healthcare professionals should work together. The approach towards interdisciplinary collaboration can improve not only the quality of research but also inter-profession communications. A similar trend was observed in this study, where most of the publications were from authors with different backgrounds, including dentistry, material sciences, microbiology, etc. The number of multiple authors could be due to the emergence of interest among researchers in the field of antibacterial adhesives. This analysis is important to highlight the role of different researchers who can contribute to the field of antibacterial adhesives in several capacities. It is expected that in the future, more collaborative work will come, and new or innovative antibacterial agents will be introduced. Furthermore, a larger number of publications have multiple authors, and the contribution of 4–6 authors in articles have the highest TP and can be considered the most productive. This can be related to the collaboration between various institutes, countries, and researchers to expedite the development of antibacterial dental adhesives. 

### 4.7. Limitations and Future Research Directions

This study has certain limitations. Albeit the researchers thoroughly tried to include all the relevant keywords to retrieve the literature, it is a possibility that few important studies might have been missed. Self-citation was not assessed in this study, as there is no standard mechanism to identify self-citation. In this study, articles indexed in Web of Science were included; other databases such as Scopus, PubMed, and Google Scholar were not included. The meeting, case report, letter, abstract, biography, editorial were not included. 

Even with the limitation, the present study on antibacterial adhesives opens further opportunities for future research. Researchers from dental material sciences can formulate strategies on the topics that are developing and have gained little attention in the previous investigations. In addition, they can find the most influential papers, authors, and journals to identify the research gaps and new insights in this area. Future research directions based on this bibliometric analysis include (1) research in antibacterial adhesive needs to focus on more targeted areas i.e., antibacterial monomers, antibacterial nanoparticles, antibacterial peptides, etc.; (2) comparative research on this topic from developed and developing countries; and (3) use of more comprehensive and broader demographic variables. 

## 5. Conclusions

The overall purpose of this study was to provide a comprehensive bibliographic review of the research published on antibacterial adhesives. The publication trend was divided into two ranges—i.e., 1996–2012 and 2013–2020—whereby a substantial increase in publications was found from 2013 onward on this topic. Most of the publications with the highest citations were mainly from the United States, specifically from the University of Maryland, USA. However, the trend showed that multiple authors worked together and published papers on this topic. The articles were mostly published in *Dental Materials*, and articles based on quaternary ammonium received the highest number of citations, whereby “quaternary ammonium”, “antibacterial adhesive”, and “antibacterial monomers” have been used commonly as keywords. It is expected that this quantitative bibliometric study will provide a direction to researchers, funding organizations, and policy makers about strength and missing gaps in the antibacterial dental adhesives field. 

## Figures and Tables

**Table 1:** Most influential countries and organizations in terms of total publications (TP), total citations (TC), and citation impact (CI)

Rank	Country	TP	TC	CI	Rank	Organizations	Country	TP	TC	CI
1	USA	99	2480	25.05	1	The University of Maryland	USA	53	1713	32.32
2	China	91	2473	27.18	2	Sichuan University	China	32	1128	35.25
3	Brazil	59	861	14.59	3	Capital Medical University	China	25	877	35.08
4	Japan	22	917	41.68	4	Fourth Military Medical University	China	24	984	41.00
5	Turkey	13	195	15.00	5	University of Maryland, Baltimore County	USA	21	932	44.38
6	Canada	8	44	5.50	6	Osaka University	Japan	16	810	50.63
7	Italy	8	137	17.13	7	Universidade Estadual De Campinas	Brazil	12	93	7.75
8	Germany	6	131	21.83	8	Federal University of Rio Grande do Sul	Brazil	11	71	6.45
9	South Korea	6	199	33.17	9	Universidade Estadual de Ponta Grossa	Brazil	9	72	8.00
10	Spain	6	281	46.83	10	National Institute of Standards and Technology	USA	7	164	23.43

**Table 2:** Highly influential journals in relation to published articles on antibacterial dental adhesives

Source	TP	TC	CI	Country	Publisher	IF	Q
*Dental Materials*	40	1468	36.70	The Netherlands	Elsevier	4.5	1
*Journal of Dentistry*	29	747	25.76	The Netherlands	Elsevier	3.24	1
*Acta Biomaterialia*	10	312	31.20	The Netherlands	Elsevier	7.24	1
*Journal of Dental Research*	10	594	59.40	USA	Sage	4.91	1
*Journal of Adhesive Dentistry*	9	119	13.22	USA	Quintessence Publishing	2.38	2
*Journal of Biomedical Materials Research Part B-Applied Biomaterials*	9	246	27.33	USA	Wiley	2.83	3
*International Journal of Adhesion and Adhesives*	8	21	2.63	The Netherlands	Elsevier	2.67	2
*Journal of The Mechanical Behavior of Biomedical Materials*	6	13	2.17	The Netherlands	Elsevier	3.37	2
*Operative Dentistry*	6	176	29.33	USA	Operative Dentistry	2.21	2
*Dental Materials Journal*	5	14	2.80	Japan	Japanese Society for Dental Materials and Devices	1.36	3

**Table 3:** Top 10 highly cited articles on antibacterial dental adhesive

Title	Author	Source	TC	C/Y	Year
Antibacterial Activity of Dental Composites Containing Quaternary Ammonium Polyethylenimine Nanoparticles Against Streptococcus Mutans	Beyth et al. [55]	*Biomaterials*	300	21.43	2006
Antibacterial Activity and Bonding Characteristics of An Adhesive Resin Containing Antibacterial Monomer MDPB	Imazato et al. [137]	*Dental Materials*	181	10.65	2003
Incorporation of Antibacterial Monomer MDPB into Dentin Primer	Imazato et al. [138]	*Journal of Dental Research*	148	11.38	1997
Experimental Antimicrobial Orthodontic Adhesives Using Nanofillers And Silver Nanoparticles	Ahn et al. [36]	*Dental Materials*	146	13.27	2009
In Vitro Antibacterial Effects of The Dentin Primer of Clearfil Protect Bond	Imazato et al. [139]	*Dental Materials*	138	9.86	2006
Effects of a Dental Adhesive Incorporating Antibacterial Monomer on The Growth, Adherence and Membrane Integrity of Streptococcus Mutans	Li et al. [160]	*Journal of Dentistry*	133	12.09	2009
Anti-Biofilm Dentin Primer with Quaternary Ammonium and Silver Nanoparticles	Cheng et al. [72]	*Journal of Dental Research*	116	14.50	2012
An In Vivo Evaluation of Bonding Ability of Comprehensive Antibacterial Adhesive System Incorporating MDPB	Imazato et al. [141]	*Dental Materials*	106	8.15	2007
Effect of Quaternary Ammonium and Silver Nanoparticle-Containing Adhesives on Dentin Bond Strength and Dental Plaque Microcosm Biofilms	Zhang et al. [269]	*Dental Materials*	98	12.25	2012
Novel Dental Adhesives Containing Nanoparticles of Silver and Amorphous Calcium Phosphate	Melo et al. [191]	*Dental Materials*	95	101	2013
